# The challenge of finding new therapeutic avenues in soft tissue sarcomas

**DOI:** 10.1186/s13569-019-0115-4

**Published:** 2019-04-10

**Authors:** Bernd Kasper

**Affiliations:** Sarcoma Unit, Interdisciplinary Tumor Center Mannheim, Mannheim University Medical Center, University of Heidelberg, Theodor-Kutzer-Ufer 1-3, 68167 Mannheim, Germany

**Keywords:** Soft tissue sarcoma, Systemic treatment, Metastatic disease, Trabectedin, Pazopanib, Eribulin

## Abstract

Soft tissue sarcomas are rare malignancies of mesenchymal origin comprising about 1% of all adult cancers. Systemic therapies for locally advanced and metastatic disease have been restricted for decades to very few effective and approved agents such as doxorubicin and ifosfamide. However, new therapeutic avenues including new drug developments and registrations such as trabectedin, pazopanib and eribulin as well as numerous clinical trial options have recently enriched the therapeutic armamentarium in the treatment of patients with advanced soft tissue sarcomas. The challenges and pitfalls of finding such new therapeutic avenues in recent years for the treatment benefit of patients with soft tissue sarcomas will be presented in this chapter within the thematic series on “Challenges in Sarcoma”.

## Background

Soft tissue sarcomas (STS) are a rare group of tumors of mesenchymal origin comprising about 1% of all malignancies in the adulthood. According to the 2013 updated World Health Organization classification STS represents a highly heterogeneous tumor entity of more than 50 subtypes showing distinct histological, molecular and certainly clinical characteristics [1]. Conventional chemotherapy with doxorubicin and/or ifosfamide still represents the backbone of systemic treatment in the locally advanced and metastatic setting sequentially or in combination [2]. Since the early 1980’s several trials have been investigating the addition of other chemotherapeutic drugs to doxorubicin in order to improve overall survival (OS). However, no statistically significant OS benefit could be demonstrated. Even in the European Organisation for Research and Treatment of Cancer (EORTC) Soft Tissue and Bone Sarcoma Group (STBSG) 62012 phase III study including 455 patients treated either with single-agent doxorubicin or with a combination regimen of doxorubicin plus high-dose ifosfamide the primary endpoint, overall survival, could not be met. Although the overall response rate (ORR) was nearly doubled (27% vs 14%) and the progression-free survival (PFS) could be significantly prolonged in the combination arm (7.4 vs 4.6 months), the doxorubicin plus ifosfamide combination did not lead to a statistically significant improvement in OS (14.3 vs 12.8 months) even though showing far more toxicity than doxorubicin alone [3]. After more than 40 years of standard first-line anthracycline therapy olaratumab, a platelet-derived growth factor receptor alpha (PDGFRα) inhibitor, in combination with doxorubicin raised hope for a new first-line therapeutic option for advanced STS patients [4]; however, results of the phase III study could not confirm the initially observed OS benefit. With the successful approval of trabectedin, pazopanib and eribulin for specific STS subtypes the treatment landscape in the clinical setting beyond first-line has been broadened and promising and well-tolerated systemic treatment options can be offered to our patients [5–8]. However, the prognosis of advanced STS patients is still unfavorable [9, 10]; median OS has increased during the last few years but is still approximately 15–18 months. Hence, there is clearly an unmet need to aim for new avenues, innovative drugs and new treatment strategies in this disease [11].

## New therapeutic avenues

Several promising agents have been investigated in recent years in large, multicentre, international registration trials. Few of them having proven efficacy were approved by the corresponding medical health authorities and reached marketing authorization. These registered compounds and new therapeutic avenues will be presented in more detail divided into (1) first-line avenues, (2) beyond first-line avenues and (3) avenues in development.

### First-line avenues

Olaratumab is a recombinant human immunoglobulin G subclass (IgG1)-type monoclonal antibody that binds to PDGFRα. PDGFRα activation by its ligand regulates cell proliferation, differentiation and survival. Olaratumab has been investigated in a phase Ib/II trial (n = 133) randomizing patients to a treatment with doxorubicin or doxorubicin combined with olaratumab. Not only PFS could be significantly prolonged by the addition of olaratumab (6.6 vs 4.1 months; HR 0.67; *p* = 0.0615), an improvement in OS of 11.8 months (26.5 vs 14.7 months; HR 0.46; *p* = 0.0003) could be detected in the combination arm for the treatment of locally advanced and metastatic STS [4]. Several limitations of the phase II trial had to be taken into account such as low patient numbers, possible favorable selection of histologies, unbalanced administration of olaratumab as maintenance therapy, etc. Toxicity has to be considered and outweighed against the uncertainty of the effect and the high costs of the drug. Based on these results the European Medicines Agency (EMA) recommended the granting of a conditional marketing authorization for olaratumab. Unfortunately, the subsequent international, multicenter, phase III ANNOUNCE study (NCT02451943) could not confirm the phase II results. The study did not meet the primary endpoint of OS in either the overall population (HR 1.05; median 20.4 vs 19.7 months for olaratumab plus doxorubicin vs doxorubicin plus placebo) or in the leiomyosarcoma sub-population (HR 0.95; median 21.6 vs 21.9 months for olaratumab plus doxorubicin vs doxorubicin plus placebo). Median PFS and ORR were reduced in patients who received the combination of olaratumab plus doxorubicin (median PFS: 5.4 vs 6.8 months; ORR: 14% vs 18.3%).

Another drug candidate which has been evaluated in the first-line treatment of STS was evofosfamide (TH-302), an investigational prodrug which is activated at very low levels of oxygen only. Tumor hypoxia is a common phenomenon in many human solid tumors like STS. Therefore, the side effect profile is expected to be lower when compared to conventional ifosfamide, which is characterized by a relevant incidence of neuro- and nephrotoxicity especially when given in high doses. The completed phase III trial (NCT01440088) was a randomized, open-label, global, multicenter, phase III study designed to assess the efficacy and safety of evofosfamide in combination with doxorubicin compared to doxorubicin alone in previously untreated patients with locally advanced, unresectable or metastatic STS. A total of 640 patients were randomized; the primary endpoint of the study was OS. The response rate was slightly better in the combination arm, 28.4% vs 18.3%, respectively. Disappointingly, no significant difference in median OS and PFS could be detected with the combination of evofosfamide and doxorubicin when compared with doxorubicin single-agent, 18.4 vs 19 months (HR 1.06) and 6.3 vs 6 months (HR = 0.85; *p* = 0.099), respectively. Interestingly, a significant improvement in OS was reported in the subgroup (n = 34) of synovial sarcomas, 22 vs 9 months (HR = 0.32), respectively, underlining the sensitivity of this STS subentity to oxazaphosphorine-based chemotherapy [12]. The most common grade 3 or worse adverse events in both treatment groups were hematological, including anemia (150 [48%] of 313 patients in the doxorubicin plus evofosfamide group vs 65 [21%] of 308 in the doxorubicin group), neutropenia (47 [15%] vs 92 [30%]), febrile neutropenia (57 [18%] vs 34 [11%]), leucopenia (22 [7%] vs 17 [6%]), decreased neutrophil count (31 [10%] vs 41 [13%]), and decreased white blood cell count (39 [13%] vs 33 [11%]). Grade 3/4 thrombocytopenia was more common in the combination group (45 [14%]) than in the doxorubicin alone group (4 [1%]), as was grade 3/4 stomatitis (26 [8%] vs 7 [2%]).

Unfortunately, the developmental story of both drug candidates illustrates how difficult it is to develop effective new treatment options in first-line therapy for advanced STS and that a doxorubicin-based regimen continuous to be the gold-standard even more than 40 years after its introduction into the treatment armamentarium.

### Beyond first-line avenues

After the registration of trabectedin in Europe in 2007 and pazopanib in 2012 for advanced STS subtypes, the results of another practice-changing trial have been published in 2016. The efficacy and safety of Eribulin, an inhibitor of microtubule dynamics, has been evaluated in comparison with dacarbazine in an international, multicenter, phase III trial. 450 patients with pretreated, locally advanced or metastatic leiomyosarcoma or adipocytic sarcoma have been included. The inclusion of these two STS subtypes originated in a treatment benefit seen in these two strata in the previously conducted phase II trial by the EORTC/STBSG [13]. The primary endpoint OS was shown to be significantly improved by 2 months (13.5 vs 11.5 months; HR 0.77; *p* = 0.0169) in favor of eribulin when compared to dacarbazine. In particular, subgroup analysis revealed an OS benefit in the liposarcoma cohort. Median OS was reported to be 15.6 months in the eribulin group versus 8.4 months in the dacarbazine treatment arm (HR 0.511; *p* = 0.0006) [8]. Based on these results, EMA approved eribulin in 2016 for the treatment of adipocytic sarcomas in patients who received prior chemotherapy containing an anthracycline regimen.

### Avenues in development

Aldoxorubicin, a tumor-targeted doxorubicin conjugate (with an acid sensitive linker), has been under investigation in recent years. The randomized phase II trial compared efficacy and safety parameters of aldoxorubicin versus doxorubicin in the first-line setting. Aldoxorubicin showed a significant prolongation of the PFS (5.6 vs 2.7 months; *p* = 0.02) and the 6-months PFS rate (46% vs 23%; *p* = 0.02). Notably, no cardiotoxicity was documented in the patients treated with aldoxorubicin [14]. The subsequent phase III study (NCT02049905), however, investigated aldoxorubicin in the second-line setting when compared to treatment at investigator’s choice (dacarbazine, pazopanib, gemcitabine plus docetaxel, doxorubicin, ifosfamide). The trial has been regularly closed after reaching the planned accrual of 433 patients. Aldoxorubicin was not able to demonstrate a statistically significant improvement regarding PFS as the primary study endpoint (4.1 vs 2.9 months; *p* = 0.087) revealing a negative study for the whole study population. However, in the subcohort of l-sarcomas (leiomyosarcomas and liposarcomas, 57.5%) aldoxorubicin could improve PFS (5.3 vs 2.9 months; *p* = 0.007) and the disease control rate (41.7 vs 27%; *p* = 0.016). Interestingly, cardiotoxicity was less documented in the aldoxorubicin arm compared to the conventional doxorubicin arm rendering this compound an interesting alternative to conventional doxorubicin [15]. Based on these favorable toxicity results, the company is planning to submit a new drug application for aldoxorubicin to the US Food and Drug Administration.

Palbociclib, a selective CDK4/CDK6-inhibitor, and DS-3032b, a MDM2-inhibitor, have both been investigated for the treatment of well- and de-differentiated liposarcomas (WDLS/DDLS). Both targets act as important negative regulators of p53, a tumor suppressor gene. Several phase I and II trials have been reported and/or published so far [16–18]. Notably, palbociclib was associated with a favorable PFS rate of 66% (90% CI 51–100%) in patients with CDK4-amplified WDLS/DDLS who had progressive disease despite systemic therapy (NCT01209598). However, no further phase II/III development has been undertaken so far with these compounds.

Selinexor, an oral selective inhibitor of nuclear exportin protein, has been studied in STS and bone sarcomas [19]. Promising results have been published for the treatment of DDLS in a phase I trial recently. Although no objective responses by RECIST 1.1 could be demonstrated, 17 patients (33%) showed durable (≥ 4 months) stable diseases (NCT01896505) [20]. Therefore, a seamless phase II/III trial named SEAL (NCT02606461) has been initiated which is currently recruiting patients with DDLS in the phase III portion (n = 245) in order to learn more about the efficacy of selinexor in this specific STS cohort.

Carotuximab (TRC105) is currently under investigation for the treatment of angiosarcomas. TRC105 is a monoclonal antibody targeting endoglin (CD105) which is expressed by tumor cells in angiosarcomas and up-regulated by VEGF-inhibition [21]. Hence, TRC105 is able to suppress angiogenesis and might enhance the activity of bevacizumab or other tyrosine multi-kinase inhibitors such as pazopanib [22]. Based on this pathomechanism a phase Ib/II trial combining TRC105 with pazopanib (800 mg daily) has been conducted (NCT01975519). A tumor reduction could be documented in five angiosarcoma patients; two of them had progressive disease on previous pazopanib therapy. Two patients with cutaneous angiosarcoma experienced a complete remission according to RECIST. Median PFS for the angiosarcoma patients was 12.9 months [23]. An adaptive population enrichment phase III trial (TAPPAS, NCT02979899) investigating the combination of carotuximab in combination with standard dose pazopanib compared to single-agent pazopanib 800 mg daily in patients with advanced angiosarcomas is currently under recruitment [24].

The revival of therapeutic affectation of the immune system has revolutionized patient outcome in many solid tumors in the last few years, in particular in melanoma [25]. This Immunotherapy approach is currently also being evaluated in STS. The largest published clinical phase II study has been performed by the Sarcoma Alliance for Research through Collaboration (SARC) study group. In total, 80 patients with STS and bone sarcomas from 12 participating centers have been treated with the PD1-inhibitor pembrolizumab. The primary endpoint was the response rate. A response rate of 18% was reported for the 40 included STS patients. The heterogeneity of STS in terms of biology and response to systemic treatment could be confirmed once again by showing different response rates depending on sarcoma subtypes. A promising response rate of 40% was reported for the undifferentiated, pleomorphic sarcoma cohort. In contrast, only 5% of bone sarcoma patients experienced a tumor response [26]. Further work has to be done in order to clarify the role of immunotherapy in STS. In particular, investigating potential predictive markers on a molecular level for suggested differences in treatment sensitivity and evaluating the optimal treatment combinations of checkpoint inhibitors with chemotherapy, radiotherapy or targeted treatment options would be of major interest.

## Commentary summary

What lessons have we learnt in drug development for sarcomas in the last few years? Since the early 1970’s systemic therapies for locally advanced and metastatic soft tissue sarcomas have been restricted to very few effective and approved agents such as doxorubicin and ifosfamide—and that hasn’t substantially changed until today. In 2007, trabectedin has been granted approval only in Europe and later in 2015 in the USA and other countries worldwide; it has turned out to be an effective compound especially for liposarcoma and leiomyosarcoma patients with the ability to stabilize the disease for long time periods. The next two registrations for STS, pazopanib and eribulin, have both been developed systematically in the phase II within the EORTC Soft Tissue and Bone Sarcoma Group (STBSG). Activity has been studied in four different strata—leiomyosarcoma, liposarcoma, synovial sarcoma and other subtypes—and further development of the drug has only been undertaken in those strata where predefined EORTC activity criteria have been met. For example, for pazopanib no activity could be demonstrated for the adipocytic stratum. Hence, liposarcoma patients were not included in the subsequent phase III PALETTE study and they are excluded from the registration label [7]. For eribulin, activity could only be demonstrated in the liposarcoma and leiomyosarcoma strata. Hence, the subsequent phase III registration trial comparing eribulin to dacarbazine only included these two histologies [8]. This approach at least takes into account the perspective of disease groups and different histologies. In contrast, a number of large phase III trials in the last few years failed (ridaforolimus, palifosfamide, and evofosfamide); exactly because disease subgroups were not taken into account and all histologies were lumped together in one trial. More and more upcoming clinical trials address specific subtypes such as selinexor in liposarcoma or carotuximab in angiosarcoma and are leading to a more “personalization of sarcoma treatment”.

What new actions can we take from these lessons? What should we not do? We should only develop drugs with a clear scientific rationale and with clearly documented preclinical and early clinical activity. Empirical use of new drugs in different entities as it currently appears to be done in the immune-oncology field should be avoided. We should restrain from lumping together all histological subtypes in one trial. Rather to ensure that reimbursement follows licensing approvals, we should aim for meaningful treatment effects outweighing possible toxicities and drug costs and including patient reported outcome measures.

What pathway are we currently on and where might we consider where we could get to? We are definitely at a current breakpoint from classical drug development to more personalized strategies taking into account sequencing techniques with the possibility to identify patients for targeted therapies. However, we are still far away from designing a kind of personalized therapy for each of our patients. But with the number of registered drugs and the possibility to sequence them one after the other—even though we do not know which sequence is the best—we have been able to significantly prolong the overall survival for our advanced STS patients in the most recent years aiming to reach a 2 years median overall survival time.

## Key points

- Patients suffering from sarcoma should be admitted to sarcoma centers early in their disease course. Sarcoma treatment should be concentrated in designated institutions with a high expertise in sarcoma diagnostics and therapy. The European Reference Network (ERN) for rare adult solid cancers (EURACAN) will certainly play a central role in this respect in the near future.
- Doxorubicin-based chemotherapy remains as the gold-standard treatment for locally advanced and metastatic STS patients in the first-line setting.
- Trabectedin, pazopanib and eribulin represent efficacious and well-tolerated treatment options beyond first-line therapy in several STS subtypes.
- Several promising new drug compounds (palbociclib, selinexor, carotuximab, etc.) and new therapeutic avenues such as immunotherapy are currently studied within ongoing clinical trials.
- Patients should preferably be treated within clinical trials, if available. International collaboration should be promoted in this matter.
